# Cross-linguistic transfer in Turkish–English bilinguals’ descriptions of motion events

**DOI:** 10.1016/j.lingua.2021.103153

**Published:** 2021-08-25

**Authors:** Samantha N. Emerson, Valery D. Limia, Şeyda Özçalışkan

**Affiliations:** a Boys Town National Research Hospital, Center for Childhood Deafness, Learning, & Language, 555 North 30th St., Omaha, NE 68131, USA; b Florida Institute of Technology, Institutional Research & Effectiveness, 150 West University Blvd., Melbourne, FL 32901, USA; c Georgia State University, Department of Psychology, 140 Decatur St., Atlanta, GA 30303, USA

**Keywords:** Typology, Bilingualism, Monolingualism, Narratives, Manner of motion, Path of motion

## Abstract

Languages differ in how they express motion: Languages like English prefer to conflate manner and path into the same clause and express both elements frequently while languages like Turkish prefer to express these elements separately, with a greater preference for the expression of path of motion. While typological patterns are well-established for monolingual speakers of a variety of languages, relatively less is known about motion expression in bilingual speakers. The current study examined the packaging (expressing each element in separate clauses or within the same clause) and lexical choices (amount and diversity of manner and path verbs) for motion expression in monolingual speakers of Turkish or English and advanced Turkish (L1)–English (L2) bilinguals in a narrative elicitation task. Bilinguals were successful in attaining many English-like patterns of expression in their L2 English but also showed some packaging and lexical choices that were intermediate between English and Turkish monolinguals—thus providing evidence of an L1-to-L2 cross-linguistic effect. Subtle effects of L2 on L1 were also found in bilinguals’ lexical choices for the expression of motion in their L1 Turkish. Altogether, our results demonstrate bi-directional transfer effects of learning a typologically distinct language in advanced Turkish–English bilinguals.

## 1. INTRODUCTION

Languages differ in how they package the *path* (the trajectory of motion) and *manner* (how a figure moves) components of motion events (Talmy, 1985, 2000). Speakers of languages such as English conflate manner and path into the same clause and frequently express both types of motion while speakers of languages such as Turkish express these components separately in multiple clauses, with path being expressed more frequently than manner (Özçalışkan et al., 2016a). The fact that cross-linguistic differences exist in the realm of motion raises an intriguing question about people who speak more than one language, whether bilingual speakers of typologically distinct languages flexibly shift their patterns of motion expression based on the language they use. While much research has been devoted to understanding motion expression in monolingual speakers of typologically different languages (e.g., Beavers et al., 2010; Özçalışkan & Slobin, 1999; Slobin, 2004; Talmy, 1985; Talmy, 2000), cross-linguistic influences in bilinguals’ expression of motion are less extensively studied. In this study, we examine motion expression in Turkish (L1)–English (L2) bilinguals in telling narratives in each language, comparing them to the monolinguals of each language.

### 1.1. Expression of motion in monolinguals

Motion in space is a basic human experience with two key components: manner that specifies how one moves (e.g., *running*, *jumping*, *crawling*, *swimming*) and path that specifies in which direction one moves (e.g., *enter*, *exit, up, down*). According to Talmy (1985, 2000), languages can be divided into two categories based on how these two components of motion are verbalized: satellite-framed (S-languages) and verb-framed (V-languages). In S-languages (e.g., English), path is typically expressed outside the verb in a particle or preposition allowing the verb to express manner (see example 1). In V-languages (e.g., Turkish), path is typically expressed within the verb. The expression of manner in these languages, on the other hand, requires the speaker to either add an optional adjunct expression (e.g., *hızla* = ‘rapidly’) or a subordinate clause with a manner verb (see example 2), resulting in lower expression of manner information in these languages.

**Table:** 

(1)	*The man* ***runs*** *into* *the house*
(2)	*Adam ev-e* (***koşarak***) *girer*
	‘man house-TO (**running**) enters’
	‘The man enters the house (**running**)’

These structural differences in motion expression lead speakers of S-languages to produce expressions where manner and path are conflated into the same clause while speakers of V-languages tend to produce separate clauses for each component, (Kita & Özyürek, 2003; Özçalışkan et al., 2016a,b). Furthermore, because manner can be expressed with relative ease in the verb in S-languages, speakers of these languages tend to produce manner verbs more frequently (i.e., tokens) and have a greater variety (i.e., types) of manner verbs than speakers of V-languages (Özçalışkan & Slobin, 1999; Slobin, 2004). For example, the V-language Turkish has only the words *atla* and *atıl* (meaning ‘to jump’ and ‘to leap’, respectively), as variants of the same manner. In contrast, the S-language English contains many more words such as *jump*, *bounce*, *hop*, *skip*, *leap*, *bound*, *pop*, *bob*, *vault*, and *spring* along with several others to express the very same concept ‘jumping’.

While this binary typology fits well with many of the world’s languages, it does not fit quite as well with some.^1^ One approach to account for these difficult-to-classify languages is to think of manner as a cline with languages ranging from low to high manner salience based on the accessibility of lexical devices for expression of manner (Slobin, 2004). Unlike manner, which is an optional component for many languages, path is considered to be obligatory for a motion expression to occur in any language (Talmy, 1985). Consequently, languages cannot be compared based on how readily expressible path is. However, languages *do* vary in how detailed their descriptions of path are (Ibarretxe-Antuñano, 2004a, 2009). For example, languages like Basque, Turkish, and Danish frequently attach one or more satellites to the main verb to describe the ground or various milestones within the trajectory of motion (Ibarretxe-Antuñano, 2009; Özçalışkan, 2004, 2009), suggesting that languages can be classified on both clines of manner-salience and path-salience. As such, a fuller account of cross-linguistic variability in motion expression should include comparisons both *within a language* to understand the relative production of manner and path information within a language of a given type and *between languages* to determine relative production of manner and path information between languages of different types.

### 1.2. Expression of motion in bilinguals

Expression of motion has been studied extensively in first language production contexts, but relatively less is known about the expression of motion in second language production contexts (cf. Cadierno, 2017 for a review). While languages can be divided typologically based on whether and how speakers choose to express manner and path information, most languages have the lexical and grammatical structures available to allow speakers express both V-language-like or S-language-like descriptions when talking about motion (Beavers et al., 2010). As such, learning L2 patterns of motion expression in a typologically distinct language poses the individual with the task of learning a new set while suppressing an old set of expression patterns—ones that are often grammatical in L2 but unnatural sounding to native ears. This often results in ‘L1-to-L2 cross-linguistic transfer’—a phenomenon known as the failure to switch to native-sounding patterns of expression in one’s L2 (Jarvis & Pavlenko, 2008; Odlin, 1989). Importantly, the effects of habitual expression are unlikely to be entirely uni-directional. Rather, prolonged immersion in an L2 is also likely to also have an impact on a speaker’s L1, resulting in ‘L2-to-L1 cross-linguistic transfer’ in patterns of motion expression (Brown & Gullberg, 2008, 2010, 2011; Hohenstein et al., 2006).

There are two main levels at which cross-linguistic transfer may occur (Hohenstein et al., 2006). First, transfer can occur at the clausal level in the packaging of components of a motion event (e.g., greater use of conflated sentence-units or lower use of separated sentence-units when speaking a V-language; Özçalışkan, 2016). Second, transfer can occur at the lexical level in the token and type diversity of lexical items expressing manner or path of motion (e.g., greater use of path verbs or lower use of manner verbs when speaking an S-language; Özçalışkan & Slobin, 1999, 2000).

With respect to packaging, previous research has shown that speakers of both S- and V-languages have difficulty adjusting their patterns of expression in their L2 to match those of native speakers. For example, while speaking in their L2 V-language (e.g., Spanish, French), S-language speakers (e.g., English, Polish) have been shown to retain their L1 preferences to conflate manner and path within the same sentence-unit rather than to express them in separate sentences (L2 Spanish: Larrañaga et al., 2011; Navarro & Nicoladis, 2005; L2 French: Soroli et al., 2012) and produce separate utterances at a lower rate than monolingual speakers of the same V-languages (Navarro Nicoladis, 2005), highlighting cross-linguistic transfer effects of L1 on L2. The opposite has been shown for L2 learners of S-languages: When speaking an L2 S-language, native V-language speakers use more separated utterances than native S-language speakers (Hohenstein et al., 2006; Stam, 2006). At the same time, some studies suggest that bilinguals are more likely to attain native-like patterns in L2 if they have higher L2 proficiency (V-language L2: Larrañaga et al., 2011; S-language L2: Özçalışkan, 2016; Stam, 2010, 2015) or if the L1-to-L2 shift involves a movement from a more specific (i.e., S-language, such as Polish or German) to a more general (i.e., V-language, such as Spanish) system of expression (e.g., Lewandowski & Özçalışkan, 2021a,b). Others have found no differences in the expression of native-like patterns in expression across adult bilinguals with different levels of proficiency in L2, particularly for native S-language speakers who are learning a V-language as L2 (Hendriks & Hickmann, 2015; Soroli et al., 2012; Navarro Nicoladis, 2005). Thus, the existing research provides at least some evidence for L1-to-L2 cross-linguistic transfer for the packaging of motion information, particularly with greater levels of proficiency or for shifts from more general to more specific systems of expression.

Transfer at the lexical level, on the other hand, refers to shifts in choices about what type of information to express and the form that the information takes. Native speakers of S-languages are accustomed to expressing motion with a manner verb and consequently use a greater number and variety of manner verbs than path verbs (Özçalışkan, 2005; Özçalışkan & Slobin, 1999; Slobin, 2004). As shown in previous work, L2 speakers of V-languages with an S-language as L1 produce greater token and type frequencies of manner verbs than path verbs in their L2 (Choi & Lantolf, 2008; Phillips, 2003) as well as a greater number of manner verbs than native speakers of the V-language ge (Navarro Nicoladis, 2005). At the same time, other studies have shown that some native S-language speakers are able to learn and effectively use V-language-like patterns of expression by producing path verbs more frequently than manner verbs even among beginner or intermediate L2 learners (Lewandowski Özçalışkan, 2021a; Soroli et al., 2012; Navarro & Nicoladis, 2005);.

Fewer studies have examined verb token and type use in native V-language speakers learning an S-language as their L2. Similar to the transfer effects seen for packaging, research on lexical production has revealed that L2 learners of V-languages frequently use fewer path satellite tokens (Li et al., 2014; Stam, 2006), fewer manner verb tokens (Brown & Gullberg, 2008; Özçalışkan & Slobin, 2000), and greater path verb tokens (Hohenstein et al., 2006) compared to native speakers of the V-language, although native-like levels of token production have been seen in bilinguals with greater levels of proficiency (Hohenstein et al., 2006; Flecken et al., 2015). Unfortunately, none of these studies examined the overall number of manner and path verb *types* produced by the L2 learners compared to monolinguals. Cross-linguistic transfer for motion verb types is arguably more difficult for L2 learners of S-languages than of V-languages, particularly for manner verb types, because learners going from a V- to an S-language face the ‘few-to-many challenge’ of translating a low number of manner words in their native language to a greater number of different manner words in their L2 (Lewandowski & Özçalışkan, 2021a). As such, the acquisition of motion verb vocabulary in learners of an L2 S-language remains an important, yet open, question.

Cross-linguistic transfer can also occur from L2-to-L1 though relatively few studies have examined this type of transfer for the expression of motion and, to date, none has examined it with respect to the packaging of motion. Hohenstein and colleagues (2006) examined native Spanish speakers (V-language) learning English (S-language) as an L2 and found that while speaking in Spanish the bilinguals produced a greater number of manner verbs than Spanish monolinguals. A second line of studies examining native speakers of Japanese (V-language) who were intermediate learners of English (Brown & Gullberg, 2008, 2010, 2011) found that while speaking in their L1(Japanese), the bilinguals produced a greater amount of path satellites per clause—a feature typical of English—than monolingual Japanese speakers (Brown & Gullberg, 2010, 2011) showing an L2-to-L1 transfer. Thus, there is tentative evidence that suggests that learning an L2 can influence speakers’ expression of motion in their L1 at least with native speakers of Spanish or Japanese (both V-languages) who have learned English (S-language) as their L2.

Altogether, the literature suggests that L2 learners face challenges when learning the patterns of expression for motion events when that L2 is from a typologically distinct category than their L1. Speakers moving from an S-language L1 to a V-language L2 may have difficulty learning to increase their use of separated constructions and path verbs and lowering their use of conflated constructions and manner verbs while speakers moving from a V-language to an S-language may face the opposite set of challenges. These challenges may be even more pronounced for this latter set of bilinguals who must learn to increase their use of a relatively more complex construction type (i.e., conflated expressions which incorporate more semantic elements) and to learn additional verbs for manner—a semantic element that is rarely expressed in any form in their L1. Importantly, none of this earlier work has examined cross-linguistic transfer on both motion packaging and motion verb usage from both a between-language (i.e., the frequency with which bilinguals use a particular motion expression relative to monolingual speakers) and within-language (i.e., bias to produce one type of expression relative to another in bilinguals compared to monolinguals) perspective. The existing research has also focused almost exclusively on L1-to-L2 transfer, with relatively few studies examining L2-to-L1 transfer.

### 1.3. Present study

In this study, we examined patterns of motion event expression in adult native speakers of Turkish (V-language) who speak English (S-language) as an L2 in comparison to monolinguals in each language with a focus on differences in the expression of manner and path both between and within languages. To date, only two studies have examined cross-linguistic transfer in Turkish-English bilinguals—both with children (Aktan-Erciyes, 2020; Aktan-Erciyes et al., 2020). The studies showed that 5-year-old children who were immersed in a rich English-as-an-L2 environment produced more manner and fewer path constructions in their L1 Turkish than their Turkish monolinguals peers, demonstrating an L2-to-L1 crosslinguistic transfer effect. This L2-to-L1 transfer effect did not extend to 7-year-olds, however, who at the time of testing, had less daily exposure to their L2 English. Furthermore, while speaking in their L2 English, 7-year-olds produced more path-only and fewer manner-only constructions than the 5-year-olds, suggesting an L1-to-L2 crosslinguistic transfer effect. Our study further expands these two studies in two important ways by looking at adult bilinguals and by including a monolingual comparison group. We know from earlier work that even by age 7, children are still in the process of learning adult-like patterns of motion expression in their L1 (Hickmann et al., 2009). As such, the amount of cross-linguistic transfer seen in these studies may have been influenced by the relative maturity of the children’s L1 motion productions. This earlier work also did not have a monolingual comparison group, making it difficult to assess the amount of L1-to-L2 cross-linguistic transfer present without a clear response baseline from English monolinguals for the particular set of stimuli used in the studies.

In the present study, we build on this earlier work by examining both monolingual and bilingual adult speakers. We also expand on previous studies on motion expression in bilinguals (Cadierno, 2017) by examining speakers’ verbal responses at the level of both the clause (motion packaging) and the verb (tokens and types) separately for patterns of expression between language groups and within each group. We define *between-language patterns* as the overall production rates for each target motion element (i.e., conflated and separated packaging, manner and path verb tokens/types). This separate focus on each motion element (as opposed to the use of proportional scores) has the benefit of identifying where speakers of a language fall relative to speakers of another language on a cline of saliency for manner and for path of motion expression. As a corollary to this, we define *within-language patterns* as the strength of speakers’ preference within a language to produce a given motion element as opposed to another (i.e., bias to produce conflated motion packaging, bias to produce manner verb tokens/types).^2^

In this study we addressed two questions: We first asked whether **monolingual speakers** of English and Turkish differ in their expression of motion, following patterns found in earlier work. We predicted that monolingual speakers of English would produce patterns of motion event descriptions similar to speakers of other S-languages while Turkish speakers would produce patterns similar to those of other V-language speakers (Ibarretxe-Antuñano, 2004a, 2009; Kita & Özyürek, 2003; Lewandowski & Özçalışkan, 2021a; Özçalışkan, 2009, 2016, Özçalışkan et al., 2016a,b; Özçalışkan & Slobin, 1999; Slobin, 1996; Slobin, 2004). More specifically, for *between-language patterns* of expression, we predicted that English monolinguals would produce more conflated and fewer separated motion sentence-units than Turkish monolinguals. Likewise, English monolinguals would produce a greater amount of manner verb tokens and types than Turkish monolinguals. For *within-language patterns*, we predicted that the motion sentence-units would be more likely to be conflated (as opposed to separated) and motion verb tokens and types would be more likely to encode manner (as opposed to path) of motion information within English monolinguals while expressions would be less likely to conflate motion or to encode manner of motion within Turkish monolinguals based on earlier work (Ibarretxe-Antuñano, 2004a, 2009; Kita & Özyürek, 2003; Lewandowski & Özçalışkan, 2021a; Özçalışkan, 2009, 2016, Özçalışkan et al., 2016a,b; Özçalışkan & Slobin, 1999; Slobin, 1996, 2004).

For **L1-to-L2 cross-linguistic transfer**, we first asked whether Turkish (L1)–English (L2) bilinguals would show English-like patterns of expression when speaking their L2 English suggesting successful acquisition or, alternatively, exhibit L1-to-L2 crosslinguistic transfer showing evidence of retention of their native Turkish-like patterns of expression. For any motion expression (i.e., packaging, verb tokens, verb types) that exhibited cross-linguistic differences between the two groups of monolingual speakers, successful between- and within-language acquisition of the target expression in English by the bilinguals would be demonstrated by a significant shift away from the patterns seen in Turkish monolinguals and no significant differences from the patterns of the English monolinguals. Moderately successful acquisition would be demonstrated by patterns of expression that are intermediate between Turkish and English monolinguals but not significantly different from either group. Unsuccessful acquisition of L2 patterns would be demonstrated by productions that are not significantly different from Turkish monolinguals but significantly different from English monolinguals (i.e., continuing to use Turkish-like patterns of expression while speaking in English).

Finally, for **L2-to-L1 cross-linguistic transfer**, we asked whether Turkish (L1)–English (L2) bilinguals would show Turkish-like patterns of expression when speaking their L1 Turkish or, alternatively, exhibit L2-to-L1 cross-linguistic transfer showing evidence that learning English had affected their preferences for the expression of motion in their native language. For any given between- or within-language pattern of expression, a strong L2-to-L1 cross-linguistic influence would be demonstrated in bilinguals’ Turkish productions by a significant shift away from the patterns of expression seen in Turkish monolinguals and no significant differences from the productions of English monolinguals (i.e., the use of English-like patterns while speaking in Turkish). A moderate L2-to-L1 influence would be demonstrated by patterns that were intermediate between the two monolingual groups without significantly differing from either. No influence would be demonstrated by a significant difference from the productions of English monolinguals but not of Turkish monolinguals.

Overall, our study provides three unique contributions: First, by examining bilinguals’ productions in both Turkish (L1) and English (L2), we can examine not only L1-to-L2 cross-linguistic transfer, but also L2-to-L1 transfer. Second, examination of patterns at both the level of clausal packaging and at the level of the verb provides a more comprehensive view of the expression of motion. Lastly, examination of both between-language and within-language variability for the three groups of speakers will identify both similarities and differences in the raw rates of production for each type of motion expression as well as the strength of their biases to produce one type of expression over another, respectively.

## 2. METHODS

### 2.1. Participants

Participants for the study included 10 monolingual English speakers (*M*_age_ = 21, *range* = 22–39, 5 females), 10 monolingual Turkish speakers (*M*_age_ = 32, *range* = 18–43, 5 females), and 10 bilinguals (*M*_age_ = 29, *range* = 22–44, 5 females). *Post hoc* observed power analyses were performed using the *powerSim()* function of the *simr* package (Green MacLeod, 2016). For each analysis, 1000 simulations were run with a = 0.05. Power was assessed for the interaction term in between-language analyses using the *anova* method and for the main effect of language group in the within-language analyses using the *lr* (Likelihood Ratio) method. Results confirmed that sample sizes provided sufficient power with power estimates ranging from 90.20% (*CI*_95%_ = 88.19%, 91.97%) to 98.90% (*CI*_95%_ = 98.90%, 99.45%).

Data from monolingual English and monolingual Turkish speakers were collected in Atlanta, USA and Istanbul, Turkey, respectively; and data from bilingual speakers were collected in Atlanta, USA. None of the monolinguals spoke another language. Bilinguals were all native Turkish speakers who were advanced but late learners of English, beginning around age *M* = 11;8 (*SD* = 0.84, *range* = 10–13). All had received English-language instruction during secondary school through college and had been living in the United States for at least four years where they spoke primarily English. All bilinguals rated their written and spoken proficiency in English as either a 5 (*n* = 9) or a 4 (*n* = 1) on a five-point, Likert-like scale where 1 represented ‘not at all well’ and 5 represented ‘very well’. Turkish-language dominance was further established using a word generation task (Lezak, 1995; Spreen & Strauss, 1998), in which bilingual participants produced as many words as they could in a minute within a given category (i.e., animals, fruits) or that began with a certain letter (i.e., ‘F’, ‘A’, ‘S’). Bilingual speakers produced a greater number of words in their L1 Turkish than in their L2 English during both the category-based word generation task (L1: *M* = 19.30, *SE* = 2.72; L2: *M* = 15.65, *SE* = 3.45; Wilcoxon *Z* = 2.35, *p* = .02, *r* = 0.53) and the letter-based word generation task (L1: *M* = 15.60, *SE* = 2.64; L2: *M* = 12.00, *SE* = 3.25; Wilcoxon *Z* = 2.65, *p* = .01, *r* = 0.59).

### 2.2. Data collection and coding

Participants were given a wordless picture book known as ‘Frog, Where Are You?’ (Meyer, 1963) and asked to view the story. After finishing the book, participants retold the story in as much detail as they could remember while being video recorded. Bilinguals retold the story twice in the same session with a 10- to 15-minute break between each production. Half of the bilinguals told the story first in Turkish and then in English while the other half told the story in the reverse order to control for possible language order effects.^3^

All videos were transcribed by native speakers of each language and segmented into sentence-units. Each sentence-unit contained at least one manner or path verb and any associated arguments (e.g., subject, secondary expressions of motion in the form of a subordinate clause or adverb, direct/indirect object, modifiers). Neutral motion verbs (e.g., *move*, *go, git* = ‘go’) were not included in analyses because they did not clearly express an explicit path or manner. We further coded each sentence-unit for packaging of motion elements as separated, where only manner or path information was conveyed (e.g., *He is crawling*, *O sürünüyor* = ‘He is crawling’, *He exited the house, Evden cıktı* = ‘He from house exited’) or as conflated where both manner and path were conveyed within a single sentence-unit (e.g., *He climbed onto the deer, Geyiğin üzerine tırmandı* = ‘He the deer’s top-toward climbed’), following earlier work (Özçalışkan, 2016; Özçalışkan et al., 2016a). Sentence-units that conveyed path in the verb and manner in a subordinate clause (e.g., *He entered the house running*, *Eve girdi kosarak* = ‘House-to entered running’), which were extremely rare in the data (3 instances across groups), were also coded as separated responses—in line with earlier work (Özçalışkan et al., 2016a).

We coded each sentence-unit at the lexical level for both verbs and secondary motion expressions (i.e., linguistic elements outside the verb such as particles, adverbs, prepositions, postpositions). For verbs, we coded the main verb of each sentence unit as expressing path (e.g., *enter*, *ascend*, *cross*) or manner (e.g., *run*, *crawl*, *jump*); and we coded each secondary motion expression also as expressing path (e.g*., into*, *içine* = ‘inside-to’, *up*, *yukarıya* = ‘up-to’) or manner (e.g., *quickly*, *hızlı bir şekilde* = ‘in a quick manner’, *on his hands and knees*, *elleri ve dizleri üzerinde* = ‘on his hands and knees’), following earlier work (Özçalışkan, 2009; Özçalışkan & Slobin, 1999, 2003). We excluded the three manner verbs that served as the verb of subordinate clauses from the lexical level analysis (i.e., *running*, *dolasarak* = ‘by-wandering’, *yuzerek* = ‘by-swimming’). Table 1 displays the unadjusted means (i.e., not adjusted for random effects) and standard deviations for production by speakers in each language group for each of the primary units of analysis.

### 2.3. Data analysis

We analyzed each question with a separate set of analyses, focusing on (1) cross-linguistic differences in monolinguals’ productions, (2) L1-to-L2 transfer by comparing the English productions of the bilinguals to the two groups of monolinguals, and (3) L2-to-L1 transfer by comparing the bilinguals’ Turkish productions to the two groups of monolinguals. Each set of analyses examined motion expression for the packaging of sentence-units and the lexicalization of the individual motion elements within the sentence-unit (verbs, secondary motion expressions). The incidence of secondary motion expression encoding manner was extremely rare (3 instances for monolinguals, 7 instances for bilinguals), preventing us from conducting meaningful comparisons on secondary motion expressions of manner. The incidence of secondary path expressions were more frequent but did not show cross-linguistic differences between the two groups of monolinguals (English: *M* = 10.80, *SE* = 1.79; Turkish: *M* = 10.30, *SE* = 1.48; *F*(1, 18) = 0.06, *p* = .814), thus eliminating the possibility of secondary path expression as a source of cross-linguistic transfer in the present study. We therefore excluded secondary motion expressions—manner or path—from all analyses. This resulted in three different primary variables of interest for each of the three sets of analyses: packaging (conflated, separated), verb tokens (manner, path), and verb types (manner, path).

For each variable of interest, we first examined the *between-language di*ff*erences* in frequency of production with linear mixed models (LMMs) using the *lmer()* function. For the LMMs (Table 2; Models 1–3) the outcome variable was the frequency of occurrence for a given motion expression. The fixed factors for each LMM included the variable of interest (i.e., packaging, verb tokens, or verb type) and group. For the first set of analyses (monolinguals), group included the productions of the Turkish monolinguals (baseline) compared to English monolinguals. For the second set of analyses (bilingual L1-to-L2 transfer), group included the bilinguals’ productions in English (baseline) compared to the productions of the two monolingual groups in each language. For the third set of analyses (bilingual L2-to-L1 transfer), group included the bilinguals’ productions in Turkish (baseline) compared to productions of the two monolingual groups in each language. All LMMs included a random by-subjects intercept to account for baseline differences in the amount of motion expressions each participant produced. Significant interactions were probed with simple main effects analyses with the *testInteractions ()* function of the *phia* library (De Rosario Martínez, 2015), and adjustments were made for multiple comparisons using the Holm-Bonferroni method (Holm, 1979). Descriptive statistics for each effect include means that have been adjusted for random effects and standard errors of the link.

We then contrasted the *within-language differences* for each group (see above) with generalized linear mixed models (GLMMs) using the *glmer()* function from the *lme4* library (Bates al., 2015) in R (R Core Team, 2021). For the GLMMs (Table 2; Models 4–6) the outcome variable was defined as the likelihood of the occurrence for a particular motion expression to be conflated for packaging (as opposed to separated) or to be manner for verb tokens and verb types (as opposed to path). Group served as the only fixed factor in all GLMMs. Descriptive statistics for each effect included the percentage of occurrence for each target motion element after adjustments for random effects and the standard error of the link. When significant, the likelihood of occurrence for a particular target motion event is reported, which can be calculated by raising the constant *e* (2.72) to the power of the coefficient (i.e., *B*) for that particular effect.

## 3. RESULTS

### 3.1. Expression of motion elements in monolinguals

#### 3.1.1. Between-language analyses

**Fig. 1.**
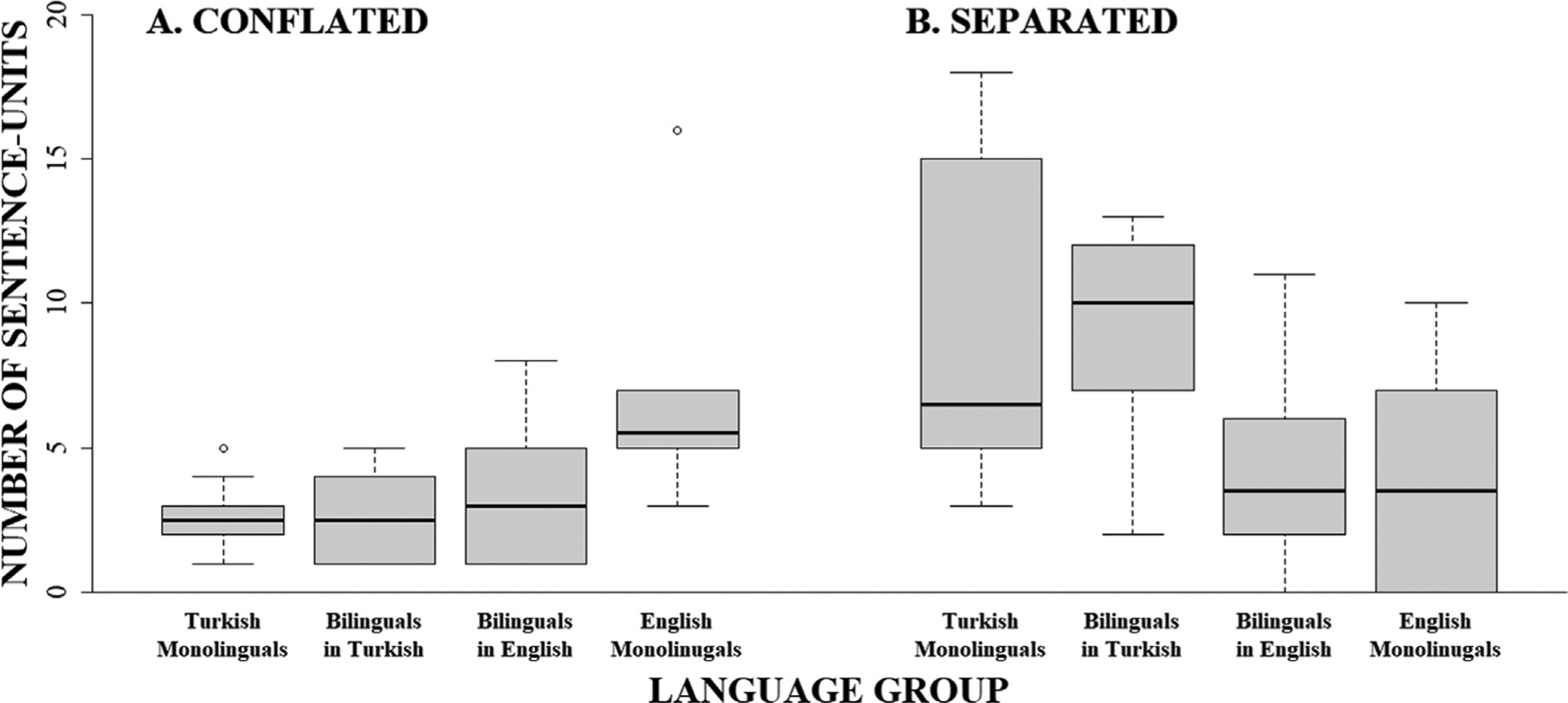
Number of conflated and separated descriptions produced by monolingual Turkish, monolingual English, and bilingual Turkish–English speakers in L1 (Turkish) and L2 (English).

We first contrasted the frequency with which monolingual speakers of either English or Turkish produced each expression of interest using linear mixed models (LMMs; Table 3). Starting first with *motion packaging* we found a significant interaction between packaging and group. As can be seen in Fig. 1,^4^ English monolinguals (*M* = 6.40, *SE* = 1.25) produced more conflated descriptions than Turkish monolinguals (*M* = 2.60, *SE* = 1.25; v^2^(1) = 4.65, *p* = .031; u = 0.34; Panel A) while Turkish monolinguals (*M* = 8.80, *SE* = 1.25) produced more separated descriptions than English monolinguals (*M* = 4.30, *SE* = 1.25; v^2^(1) = 6.53, *p* = .021; u = 0.40; Panel B).

**Fig. 2.**
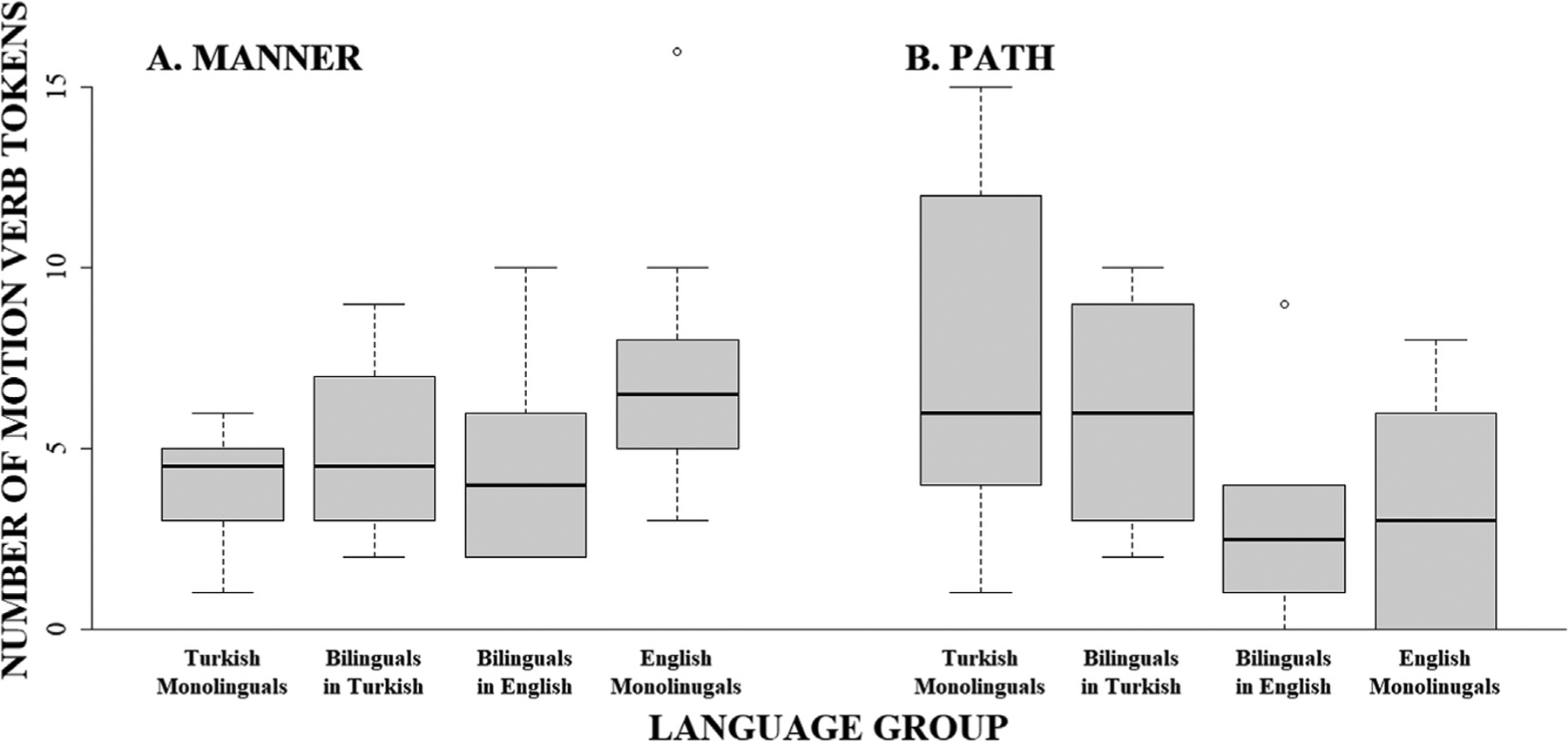
Number of manner and path verb tokens produced by monolingual Turkish, monolingual English, and bilingual Turkish–English speakers in L1 (Turkish) and L2 (English).

**Fig. 3.**
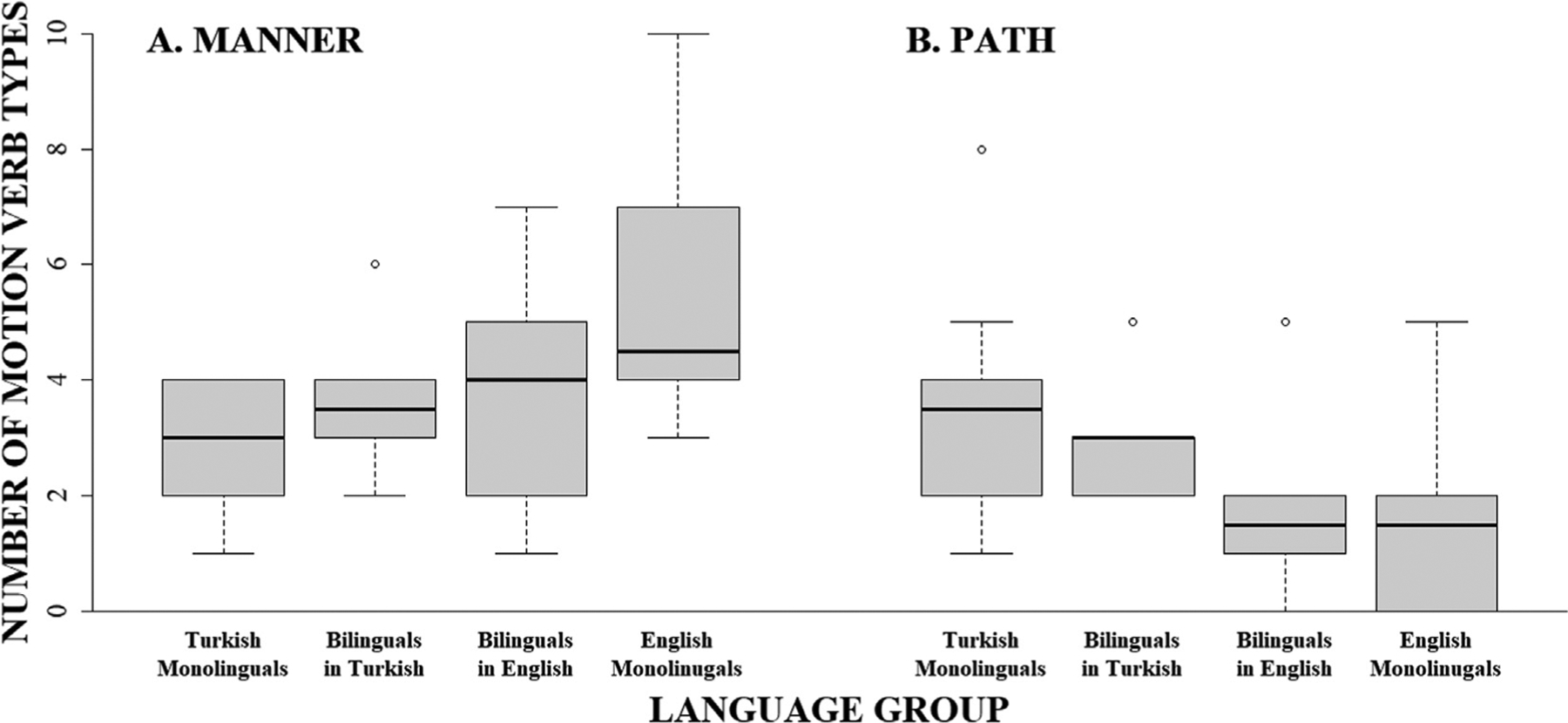
Number of manner and path verb types produced by monolingual Turkish, monolingual English, and bilingual Turkish–English speakers in L1 (Turkish) and L2 (English).

Turning next to the verbs, similar patterns were found for both the frequency of *motion verb types* and *motion verb tokens*, each showing significant interactions with group. As can be seen in Fig. 2, English monolinguals produced a greater number of manner verb tokens (*M* = 7.30, *SE* = 1.07) than Turkish monolinguals (*M* = 4.10, *SE* = 1.07, v^2^(1) = 4.46; *p* = .035; u = 0.33; Panel A) while Turkish monolinguals produced a greater number of path verb tokens (*M* = 7.30, *SE* = 1.07) than English monolinguals (*M* = 3.40, *SE* = 1.07, v^2^(1) = 6.62; *p* = .020; u = 0.41; Panel B). Likewise, as illustrated by Fig. 3, for verb types, English monolinguals produced a greater number of manner verb types (*M* = 5.40, *SE* = 0.60) than Turkish monolinguals (*M* = 2.80, *SE* = 0.60, v^2^(1) = 9.35; *p* = .004; u = 0.48; Panel A) while Turkish monolinguals produced a greater number of path verb types (*M* = 3.50, *SE* = 0.60) than English monolinguals (*M* = 1.80, *SE* = 0.60, v^2^(1) = 4.00; *p* = .046; u = 0.32; Panel B).

#### 3.1.2. Within-language analyses

**Fig. 4.**
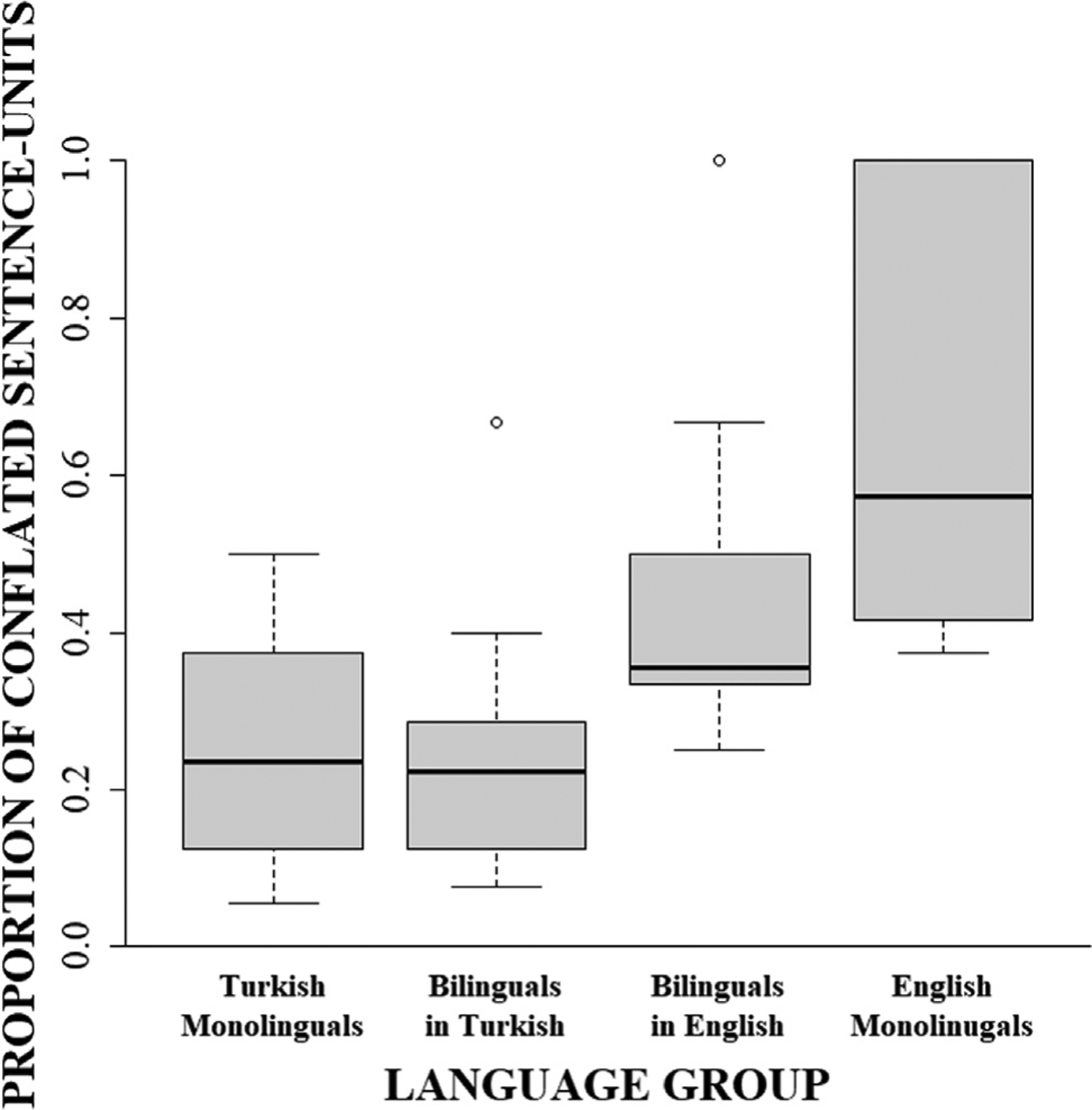
Proportion of sentence-units that conflate motion out of the total number of motion sentence-units in monolingual Turkish, monolingual English, and bilingual Turkish–English speakers in L1 (Turkish) and L2 (English).

We next examined the likelihood that speakers would produce a given expression (i.e., conflated packaging, manner verbs) and contrasted the strength of that preference between the two groups of monolinguals using generalized linear mixed models (GLMMs; Table 4). Beginning with *motion packaging*, we tested the likelihood of each motion sentence-unit having conflated packaging as opposed to separated packaging and contrasted the strength of this likelihood between monolingual speakers of each language. Our results showed an effect of group; as can be seen in Fig. 4, the sentence-units produced by monolingual English speakers (65.12%, *SE* = 0.37) were 6.24 times more likely to have a conflated packaging than monolingual Turkish speakers (22.96%, *SE* = 0.36).

**Fig. 5.**
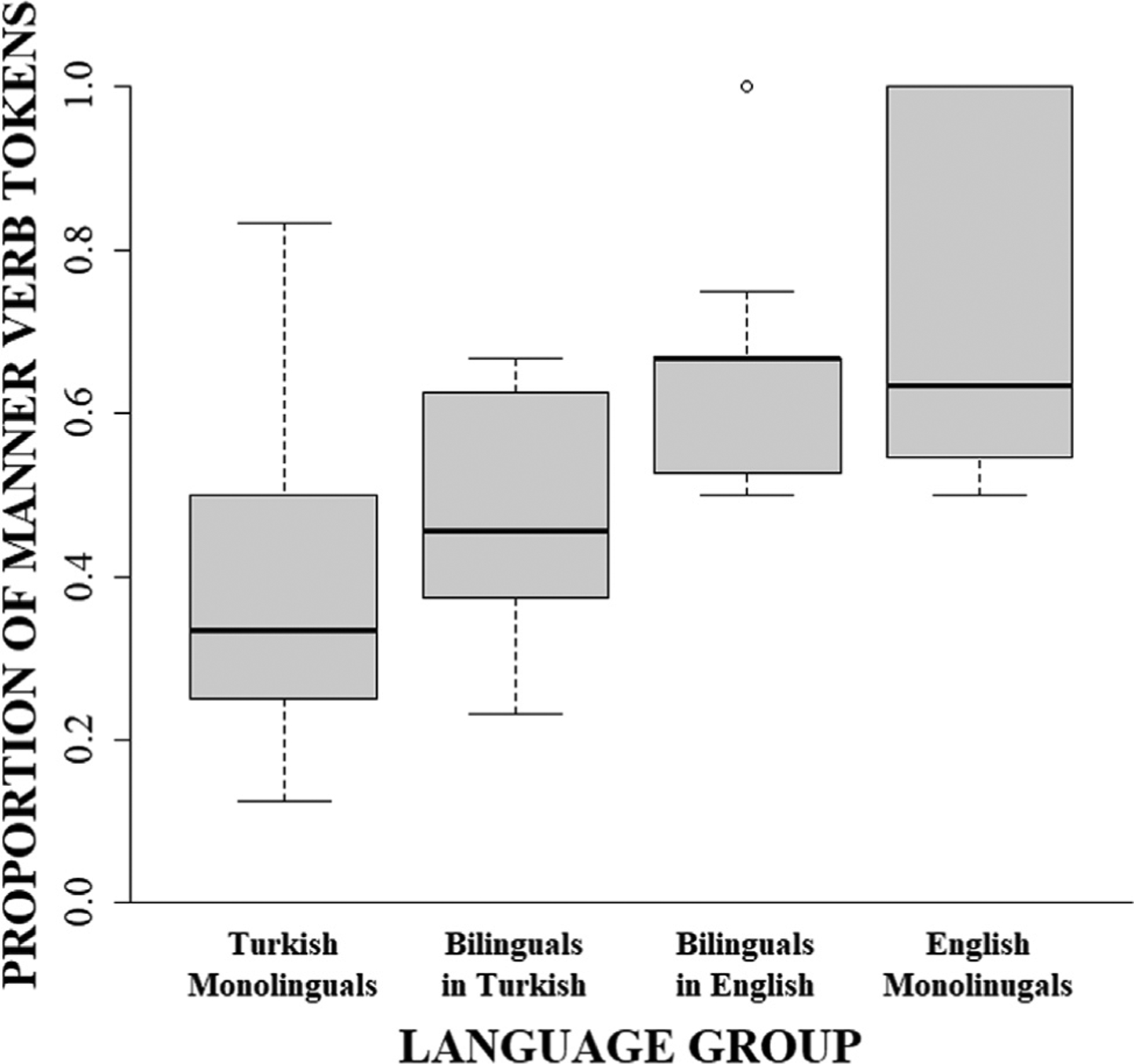
Proportion of verb tokens that encode manner of motion out of the total number of motion verb tokens in monolingual Turkish, monolingual English, and bilingual Turkish–English speakers in L1 (Turkish) and L2 (English).

**Fig. 6.**
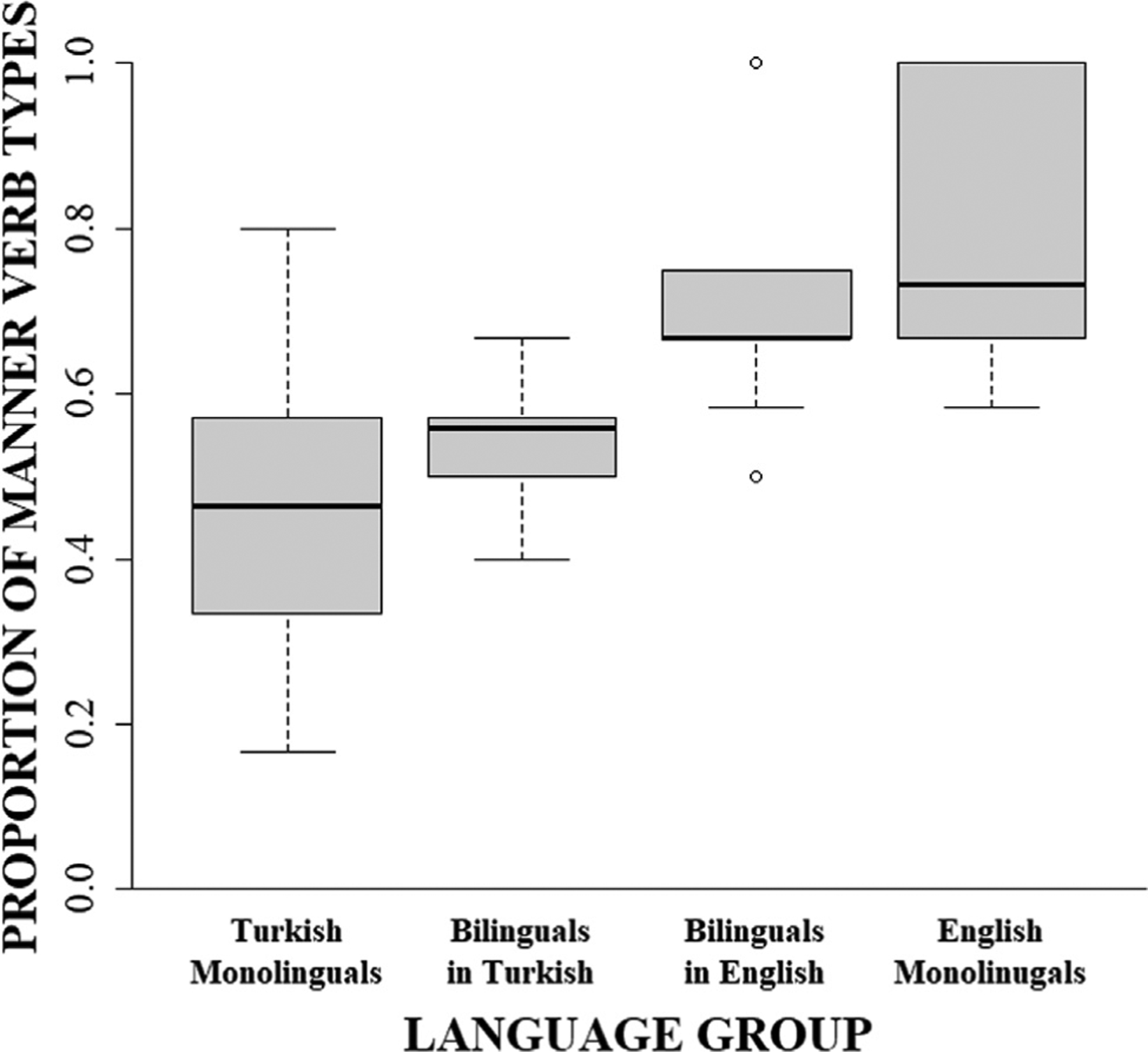
Proportion of verb types that encode manner of motion out of the total number of motion verb types in monolingual Turkish, monolingual English, and bilingual Turkish–English speakers in L1 (Turkish) and L2 (English).

Similar cross-linguistic differences were found for the likelihood of each *motion verb token* and *motion verb type* expressing manner information as opposed to path. As can be seen in Figs. 5 and 6, respectively, significant group effects revealed that motion verb tokens were 4.06 times more likely to describe manner and motion verb types were 3.75 times more likely to describe manner in English monolinguals (Tokens: 70.09%, *SE* = 0.28; Types: 75.00%, *SE* = 0.27) than in Turkish monolinguals (Tokens: 36.65%, *SE* = 0.26; Types: 44.44%, *SE* = 0.25).

Overall, results showed strong differences between monolingual speakers of the two languages. English speakers preferred to conflate manner and path into a single clause, using both a greater number and variety of manner verbs. In contrast, monolingual Turkish speakers preferred to express the two motion types in separate clauses, using a greater number and variety of path verbs.

### 3.2. L1-to-L2 cross-linguistic transfer in bilinguals

#### 3.2.1. Between-language analyses

We next examined the cross-linguistic effects that bilinguals’ L1 (Turkish) had on their motion expression in L2 (English) using LMMs (Table 5). Beginning first with *motion packaging*, we first assessed how frequently bilinguals produced each motion packaging type in English compared to monolinguals. Results revealed a significant interaction between packaging and group. As can be seen in Fig. 1, bilinguals’ use of conflated descriptions in English (L2; *M* = 3.50, *SE* = 1.15) did not differ from that of Turkish monolinguals (*M* = 2.60, *SE* = 1.15; v^2^(1) = 0.31, *p* = 1.000; u = 0.09) or English monolinguals (*M* = 6.40, *SE* = 1.15; v^2^(1) = 3.20, *p* = .221; u = 0.28; Panel A), suggesting a moderate success in approximating rates of conflated packaging in L2 English. The bilinguals’ use of separated descriptions in English (L2; *M* = 4.10, *SE* = 1.15) was also lower than Turkish monolinguals (*M* = 8.80, *SE* = 1.15; v^2^(1) = 8.40, *p* = .022; u = 0.46) and did not differ from English monolinguals (*M* = 4.30, *SE* = 1.15; v^2^(1) = 0.02, *p* = 1.000; u = 0.02; Panel B), suggesting that bilinguals were successful at reducing their overall production of separated descriptions to match that of English monolinguals.

Turning next to the verbs, the frequencies for *motion verb types* and *motion verb tokens* both showed significant interactions with group. As can be seen in Fig. 2, bilinguals’ production of manner verb tokens in English (L2; *M* = 4.80, *SE* = 1.00) was not significantly different from English monolinguals (*M* = 7.30, *SE* = 1.00; v^2^(1) = 3.10, *p* = .235; u = 0.28) or Turkish monolinguals (*M* = 4.10, *SE* = 1.00; v^2^(1) = 0.24, *p* = 1.000; u = 0.08; Panel A), suggesting a moderate success in approximating native-like rates of expression for manner verb tokens. In contrast, for path verb tokens in L2 English, bilinguals (*M* = 2.80, *SE* = 1.00) did not differ from English monolinguals (*M* = 3.40, *SE* = 1.00; v^2^(1) = 0.18, *p* = 1.000; u = 0.07) but produced fewer path verb tokens than Turkish monolinguals (*M* = 7.30, *SE* = 1.00; v^2^(1) = 10.04, *p* = .009; u = 0.50; Panel B), suggesting that the bilinguals were able to successfully lower their rates of path verb token production to levels similar to English monolinguals.

Likewise, for manner verb types (Fig. 3), bilinguals’ English productions contained an intermediate amount of verb types (*M* = 3.90, *SE* = 0.58) that was not significantly different from that of English monolinguals (*M* = 5.40, *SE* = 0.58; v^2^(1) = 3.39, *p* = .197; u = 0.29) or Turkish monolinguals (*M* = 2.80, *SE* = 0.58; v^2^(1) = 1.82, *p* = .354; u = 0.21; Panel A), suggesting a moderate success in increasing the diversity of their manner verb types. In contrast to path verb tokens, bilinguals’ English production of path verb types (*M* = 1.70, *SE* = 0.58) revealed no significant differences compared to the productions of either the English monolinguals (*M* = 1.80, *SE* = 0.58; v^2^(1) = 0.02, *p* = .902; u = 0.02) or the Turkish monolinguals (*M* = 3.50, *SE* = 0.58; v^2^(1) = 4.88, *p* = .136; u = 0.35; Panel B), suggesting a moderate success in the production of path verb types at levels similar to English monolinguals when speaking L2 English.

#### 3.2.2. Within-language analyses

We next examined whether there were differences between the likelihoods that bilinguals would produce a given type of expression in English compared to monolingual Turkish speakers or monolingual English speakers using GLMMs (Table 6). Beginning with *packaging*, we tested the likelihood that a motion sentence-unit would conflate manner and path information into the same sentence-unit (as opposed to expressing each in separated descriptions). Results revealed that sentence-units produced by the bilinguals in English (L2; 45.80%, *SE* = 0.34) were 2.80 times more likely to use a conflated packaging than Turkish monolinguals (23.09%, *SE* = 0.33) and were comparable to the productions of English monolinguals (63.95%, *SE* = 0.33), suggesting that the bilinguals were successful in learning to use conflated rather than separated expressions in English at rates similar to English monolinguals.

Turning next to the bilinguals’ verb productions in L2 English, we examined the likelihood of a motion verb token or type to express manner (as opposed to path) information and contrasted the strength of this manner-bias to the productions of monolingual Turkish and English speakers. As can be seen in Fig. 5, bilinguals’ *motion verb tokens* in English were 3.13 times more likely to describe manner of motion (64.12%, *SE* = 0.28) than Turkish monolinguals’ verb tokens (36.41%, *SE* = 0.23) and were comparable to English monolinguals’ verb tokens (69.16%, *SE* = 0.25). Likewise, as is illustrated in Fig. 6, bilinguals’ *motion verb types in L2 English w*ere 2.87 times more likely to describe manner of motion (69.64%, *SE* = 0.29) than Turkish monolinguals’ productions (44.44%, *SE* = 0.25) and were comparable to English monolinguals’ productions (75.00%, *SE* = 0.27). Thus, bilinguals were successful in learning to increase their proportional use of manner verbs tokens and types to levels similar to those used by English monolinguals.

Overall, the bilingual speakers in our sample were largely successful in gaining target L2-like patterns of expression in English, at rates roughly comparable to monolingual speakers of English. At the same time, bilinguals also showed some difficulty in increasing their production of conflated descriptions and token and type diversity of manner verbs, but this was accompanied by lower production of separated descriptions and tokens of path verbs when speaking L2 English—thus aligning with production patterns of monolingual English speakers.

### 3.3. L2-to-L1 cross-linguistic influence in bilinguals

#### 3.3.1. Between-language analyses

We last examined cross-linguistic effects bilinguals’ L2 (English) had on their L1 (Turkish), using LMMs (Table 7). Starting with *packaging*, we first assessed how frequently the bilinguals produced each type of motion packaging in Turkish compared to monolinguals and found a significant interaction between packaging and group. As can be seen in Fig. 1, bilinguals’ conflated responses in Turkish (L1; *M* = 2.60, *SE* = 1.15) were comparable to those of Turkish monolinguals (*M* = 2.60, *SE* = 1.15; v^2^(1) = 0.00, *p* = 1.000; u = 0.00) but marginally (but not significantly) lower than those of English monolinguals (*M* = 6.40, *SE* = 1.15; v^2^(1) = 5.44, *p* = .079; u = 0.37; Panel A).^5^ Likewise, bilinguals’ production of separated utterances in Turkish (L1; *M* = 8.80, *SE* = 1.15) did not differ from that of Turkish monolinguals (*M* = 8.80, *SE* = 1.15; v^2^(1) = 0.00, *p* = 1.000; u = 0.00) but was significantly greater than that of English monolinguals (*M* = 4.30, *SE* = 1.15; v^2^(1) = 7.63, *p* = .034; u = 0.44; Panel B). These results thus suggest the lack of an L2-to-L1 cross-linguistic transfer for packaging of motion elements in bilinguals’ productions in L1 Turkish.

Turning next to the verbs, the frequencies for *motion verb tokens* showed a significant interaction with group (Fig. 2). While the two groups of monolinguals were shown to differ in the frequency with which they produced both manner and path verb tokens (see Section 3.1.1.), bilinguals’ production of manner verb tokens in L1 Turkish (*M* = 5.20, *SE* = 1.01) was not significantly different from that of either Turkish monolinguals (*M* = 4.10, *SE* = 1.01; v^2^(1) = 0.60, *p* = .879; u = 0.12) or English monolinguals (*M* = 7.30, *SE* = 1.01; v^2^(1) = 2.18, *p* = .420; u = 0.23; Panel A). Likewise, bilinguals’ production of path verb tokens in L1 Turkish (*M* = 6.20, *SE* = 1.01) was not significantly different from either Turkish monolinguals’ (*M* = 7.30, *SE* = 1.01; v^2^(1) = 0.60, *p* = .879; u = 0.12) or English monolinguals’ (*M* = 3.40, *SE* = 1.01; v^2^(1) = 3.87, *p* = .196; u = 0.31; Panel B) productions. Thus, this intermediate performance between the two groups of monolinguals suggests a moderate L2-to-L1 cross-linguistic influence on verb production based on the similarity in token production of manner and path verbs between English monolinguals and bilinguals in L1 Turkish.

Likewise, the frequencies for *motion verb types* showed a significant interaction with group (Fig. 3). Again, while monolinguals’ productions differed in the variety of manner and path verb types produced (see Section 3.1.1.), bilinguals’ production of manner verb types in L1 Turkish (*M* = 3.50, *SE* = 0.53) did not differ from that of Turkish monolinguals (*M* = 2.80, *SE* = 0.53; v^2^(1) = 0.89, *p* = .694; u = 0.15) and were marginally (but not significantly) different from that of English monolinguals (*M* = 5.40, *SE* = 0.53; v^2^(1) = 6.52, *p* = .053; u = 0.40; Panel A), thus failing to provide sufficient support for an effect of L2 English on of manner verb types in L1 Turkish. By contrast, bilinguals’ production of path verb types in L1 Turkish (*M* = 2.90, *SE* = 0.53) did not differ from either monolingual Turkish (*M* = 3.50, *SE* = 0.53; v^2^(1) = 0.65, *p* = .694; u = 0.13) or monolingual English (*M* = 1.80, *SE* = 0.53; v^2^(1) = 2.19, *p* = .418; u = 0.23; Panel B) speakers’ production, suggesting a moderate cross-linguistic effect of L2 on L1, based on the similarity in type production of path verbs between English monolinguals and bilinguals in L1 Turkish.

#### 3.3.2. Within-language analyses

We next examined whether learning English had an effect on the likelihood that a bilingual would produce a given type of expression in Turkish compared to monolingual Turkish speakers or monolingual English speakers using GLMMs (Table 8). Beginning first with *motion packaging*, we tested the likelihood that a motion sentence-unit would conflate both motion elements (as opposed to expressing each in separate sentence-units). We found in L1 Turkish, bilinguals’ production of sentence-units (22.03%, *SE* = 0.32) was comparable to Turkish monolinguals (23.12%, *SE* = 0.32) but were 6.23 times less likely to have conflated descriptions than English monolinguals (63.67%, *SE* = 0.32), suggesting no L2-to-L1 cross-linguistic transfer on the likelihood of sentence-units to have a conflated packaging of motion elements.

Turning next to the bilinguals’ Turkish verb productions, we examined the likelihood that a motion verb token or type would express manner (as opposed to path) information, contrasting the strength of bilinguals’ Turkish productions with that of the two monolingual groups. As can be seen in Fig. 5, bilinguals’ *motion verb tokens* in Turkish (45.66%, *SE* = 0.22) did not significantly differ from Turkish monolinguals’ (36.38%, *SE* = 0.23) and were 2.66 times less likely to express manner of motion than English monolinguals’ (69.06%, *SE* = 0.24), suggesting lack of an L2-to-L1 cross-linguistic influence on the likelihood of verb tokens to express manner. Likewise, as is illustrated in Fig. 6, bilinguals’ *motion verb types* in Turkish (54.69%, *SE* = 0.25) were 2.49 times less likely to describe manner of motion than English monolinguals’ (75.00%, *SE* = 0.27) but were comparable to Turkish monolinguals (44.44%, *SE* = 0.25), suggesting no L2-to-L1 cross-linguistic transfer effects on the likelihood of verb types to express manner.

Overall, results revealed that L2 English had subtle effects on the bilinguals’ descriptions of motion while speaking in their L1 Turkish—mostly evident in speakers’ use of motion verbs. In particular, bilinguals, when speaking Turkish, showed moderate shifts in their verb choices, raising the amount (i.e., tokens) of manner verbs and lowering the amount and diversity (i.e., types) of path verbs to levels that were similar to that of English monolinguals. In contrast, no L2-to-L1 cross-linguistic transfer effects were observed for bilinguals while speaking in L1 Turkish for the packaging of motion events, the amount of manner verb types, or in the likelihood that their sentences would be conflated or that their verbs would express manner information.

## 4. DISCUSSION

In this study, we examined the patterns of motion expression in native Turkish speakers who had learned English as an L2 in comparison to monolingual speakers of Turkish or English—two typologically distinct languages using a narrative picture task (i.e., Frog story). Analyses of the packaging of manner and path information at the sentence-unit level and the lexicalization of motion information in the verb were used to examine cross-linguistic differences between monolingual speakers of Turkish and English, L1-to-L2 crosslinguistic transfer from Turkish to English and L2-to-L1 cross-linguistic transfer from English to Turkish. The narratives produced by monolingual speakers of English and Turkish were consistent with Talmy’s (1985, 2000) typology and previous examinations of the expression of motion events in speakers of S- and V-languages (Ibarretxe-Antuñano, 2004a, 2009; Kita & Özyürek, 2003; Lewandowski & Özçalışkan, 2021a; Özçalışkan, 2009, 2016, Özçalışkan et al., 2016a,b; Özçalışkan & Slobin, 1999; Slobin, 1996, 2004). More specifically, results demonstrated that monolingual English speakers had pronounced between- and within-language preferences for the conflated packaging and to use a greater amount (tokens) and diversity (types) of manner verbs while Turkish monolinguals showed the opposite pattern of preferences. Our analysis also showed evidence for moderate L1-to-L2 cross-linguistic transfer as well as L2-to-L1 transfer, suggesting a bi-directional effect on the patterns of motion expression used by advanced bilingual speakers of the two typologically distinct languages.

### 4.1. L1-to-L2 cross-linguistic transfer

Mastery of an L2 is not an easy task. It requires more than an understanding of the grammatical packaging and lexicon of a language; it also requires making choices between grammatical and lexical items that ‘sound right’ to a native ear. This is particularly difficult for bilinguals’ whose native language shows preferences that are in opposition to those of their L2, as is the case with the Turkish (L1)–English (L2) bilinguals in this study. Given the aforementioned results with monolinguals, to achieve native-like fluency in English, the bilinguals had to adjust their packaging of motion information to have a higher number of expressions that conflated manner and path and to switch from expressing path more frequently than manner in the verb to the opposite pattern. Importantly, this transition may have been particularly difficult for the Turkish–English bilinguals who were switching from a V-language to an S-language, a pattern shown to be evident in other V- to S-language transitions (e.g., Brown & Gullberg, 2008; Hohenstein et al., 2006; Lewandowski & Özçalışkan, 2021a; Li et al., 2014; Stam, 2006): English speakers are more likely than Turkish speakers to use conflated packaging, which includes both manner and path elements combined tightly within the bounds of a single clause. This, in turn, results in a greater density of semantic motion information for that clause than in the separated packaging that Turkish speakers strongly prefer, in which only one motion element is expressed per clause—be it a main or subordinate clause. Furthermore, the bilinguals in this study were also faced with the few-to-many problem of switching from a low amount and diversity manner verbs to the much higher levels that are typical of English monolinguals.

Overall, results from the within-language analyses revealed that the bilinguals in our study had been largely successful at acquiring English as an L2, showing a similar likelihood as monolingual English speakers to produce the conflated packaging, manner verb tokens, and manner verb types (relative to separated packaging or path verb tokens or types). However, between-language contrasts revealed that there was still some influence of the bilinguals’ L1 Turkish on their L2 English. In line with predictions concerning the grammatical complexity of conflating motion and the few-to-many transfer problem for manner verbs, bilinguals’ productions for the conflated packaging, manner verb tokens, and manner verb types was intermediate between the two groups of monolinguals, suggesting that the bilinguals were only moderately successful in acquiring these English-like patterns of expression. In contrast, bilinguals were much more successful in lowering their productions of more Turkish-like expressions, which included reduced rates of separated packaging and path verb tokens. Interestingly, bilinguals only showed a moderate decrease in their use of path verb types, suggesting that the bilinguals retained some influence of their L1 while describing path of motion with a greater variety of verbs while speaking in their L2 English.

One possible explanation for the L1-to-L2 transfer seen here is that the bilinguals in this study began to acquire English after the ‘sensitive period’—a stage of development ending around age four in which the ability to fully acquire second language patterns is negatively related to age (Meisel, 2011). Late bilinguals may be more susceptible to lexical and grammatical transfer in their L2 because their L2 exposure does not begin until after their L1 linguistic patterns have become habitual, making it difficult to fully acquire L2 patterns that are dissimilar from their L1 (Birdsong & Molis, 2001; Haugen, 1950; Slobin, 1996). To this point, L1 transfer to L2 is more commonly reported in late bilinguals (Brown & Gullberg, 2008, 2010, 2011; Hohenstein et al., 2006; Kroll & Stewart, 1994; Pavlenko & Jarvis, 2002)—a pattern that also becomes evident in our study. Future research on differences in L1-to-L2 crosslinguistic transfer between early and late bilinguals is needed, however, to further identify whether the L1 effects reported here are more malleable if the L2 is acquired during or before the sensitive period.

### 4.2. L2-to-L1 cross-linguistic transfer

Given that the patterns in a native language are well established by adulthood, relatively little research has been conducted on how the learning of an L2 might affect expression in L1. However, there is some evidence that speakers’ L1 patterns of motion expression do shift after learning an L2 (Brown & Gullberg, 2008, 2010, 2011; Hohenstein et al., 2006). As such, our final analysis compared bilinguals’ narratives in Turkish to the narratives of monolingual speakers of Turkish or English with the goal to determine whether learning English had an effect on speakers’ L1.

Within-language contrasts revealed that bilinguals remained unaffected by their L2 English in increasing their likelihood of producing the conflated packaging, manner verb types, or manner verb tokens. Likewise, between-language contrasts provided either insufficient or no evidence of L2-to-L1 cross-linguistic transfer on the way bilinguals’ packaged motion elements or in the diversity of the manner verbs they produced while speaking in L1 Turkish. However, the bilinguals did show moderate increases in their manner verb tokens and a moderate decrease in their token and type diversity of path verbs. Thus, results revealed subtle effects of the bilinguals’ L2 English on their L1 Turkish productions, especially for the lexicalization of motion in the verb.

Two factors may have worked in combination to lessen any potential L2-to-L1 cross-linguistic transfer from occurring in motion packaging: First, as aforementioned neither Turkish bilinguals speaking in English nor English monolinguals showed a strong within-language preference for conflated versus separated clauses in their narratives. In contrast, Turkish monolinguals showed a very clear preference to use separated rather than conflated packaging. Thus, the within-language bias for conflating manner and path was relatively weak compared to the Turkish bias for expressing the motion elements separately. A second factor could be that the added semantic density of conflated clauses may place an additional burden on the listener when both types of motion information are not expected (Emerson et al., 2020), as is typically the case in Turkish. As such, while speaking in Turkish, bilinguals may have continued to prefer to use the more parsimonious separated clauses, encoding only the most relevant aspect of the motion event per clause to ease the processing demands on the interlocutor. In combination, these two factors may have strengthened the *status quo* for Turkish patterns of expressions in the bilinguals, consequently preventing L2-to-L1 cross-linguistic transferal for packaging.

### 4.3. Conclusion

Altogether, our results demonstrated the predicted cross-linguistic patterns between monolingual speakers of Turkish and English: Turkish monolinguals preferentially tended to express path information and package motion information in separate clauses while English monolinguals preferentially expressed manner with a high rate of conflated clauses. Bilinguals showed multiple successes in reaching native-like patterns of expression for motion events. However, they also exhibited moderate effects of L1-to-L2 cross-linguistic transfer with respect to their productions of the conflated packaging, manner verb tokens, and manner and path verb types. Finally, bilinguals also showed subtle effects of L2-to-L1 cross-linguistic transfer in their L1 Turkish for their production of manner and path tokens and path verb types.

Our findings speak to the relationship between an established L1 and an advanced but developing L2 in the domain of motion expression in bilingual speakers of Turkish and English. Our data show that special attention needs to be paid to motion verbs in L2 that have low frequency correlates in L1 for Turkish–English bilinguals and speakers of similarly structured languages. As such, acquiring a typologically distinct L2 may require more intentional effort to reach native-like levels in late bilinguals.

## Figures and Tables

**Table 1:** Mean speech production by monolinguals in English or Turkish and bilinguals in each of their two languages.

	Sentence-Units		Packaging		Verb Tokens		Verb Types	
	Total	Motion	Conflated	Separated	Manner	Path	Manner	Path
Turkish Monolinguals	46.50 (15.52)	11.40 (5.36)	2.90(1.20)	8.50(5.56)	4.10 (1.66)	7.30 (4.52)	2.80 (1.14)	3.50 (2.07)
Bilinguals in Turkish	54.30 (17.28)	11.40 (4.33)	2.90(1.73)	8.50(3.81)	5.20 (2.39)	6.20 (3.01)	3.50 (1.18)	2.90 (0.88)
Bilinguals in English	47.10 (17.60)	7.60 (4.99)	3.70(2.58)	3.90(3.18)	4.80 (2.82)	2.80 (2.57)	3.90 (1.91)	1.70 (1.34)
English Monolinguals	48.50 (15.61)	10.70 (4.76)	6.50(3.60)	4.20(3.88)	7.20 (3.68)	3.50 (3.21)	5.40 (2.32)	1.80 (1.87)

Unadjusted means with standard deviations in parentheses.

**Table 2:** Equations for statistical models.

(1)	Packaging LMM	Freq ~ Packaging * Group + (1|Subject)
(2)	Verb Tokens LMM	Freq ~ VerbToken * Group + (1|Subject)
(3)	Verb Types LMM	Freq ~ VerbType * Group + (1|Subject)
(4)	Packaging GLMM	Conflated ~ Group + (1|Subject)
(5)	Verb Tokens GLMM	Manner ~ Group + (1|Subject)
(6)	Verb Types GLMM	Manner ~ Group + (1|Subject)

**Table 3:** LMM results for the between-language contrasts of Turkish monolinguals compared to English monolinguals.

	SumSq	MeanSq	NumDF	Den*DF*	F-Value	*p*
**Motion Packaging**						
Packaging	42. 03	42.03	1	36	2.71	0.108
Group	1.23	1.23	1	36	0.08	0.780
Packaging × Group	172.23	172.23	1	36	11.10	0.002**
**Motion Verb Tokens**						
VerbToken	1.23	1.23	1	18	0.12	0.732
Group	0.97	0.97	1	18	0.10	0.761
VerbToken × Group	126.03	126.03	1	18	12.45	0.002**
**Motion Verb Types**						
VerbType	21.03	21.03	1	18	6.85	0.017*
Group	1.50	1.50	1	18	0.49	0.494
VerbType × Group	46.23	46.23	1	18	15.06	0.001**

‘***’ *p* < .001,

‘**’ *p* < .01,

‘*’ *p* < .05,

‘.’ *p <* .10.

**Table 4:** GLMM results for the within-language contrasts of Turkish monolinguals compared to English monolinguals.

	*B*	*SE*	Wald’s *Z*	*p*
**Motion Packaging**				
Intercept	−1.21	0.36	−3.37	<0.001***
Group_English Monolinguals_	1.83	0.52	3.56	<0.001***
**Motion Verb Tokens**				
Intercept	−0.55	0.26	−2.10	0.036*
Group_English Monolinguals_	1.40	0.38	3.66	<0.001***
**Motion Verb Types**				
Intercept	−0.22	0.25	−0.88	0.379
Group_English Monolinguals_	1.32	0.37	3.55	<0.001***

* *p* < .05,

** *p* < .01,

*** *p* < .001.

**Table 5:** LMM results for the between-language contrasts of bilinguals’ English productions compared to Turkish monolinguals and English monolinguals.

	SumSq	MeanSq	Num*DF*	Den*DF*	F-Value	*p*
**Motion Packaging**						
Packaging	36.82	36.82	1	54	2.80	0.100
Group	40.90	20.45	2	54	1.56	0.220
Packaging × Group	179.23	89.62	2	54	6.82	0.002**
**Motion Verb Tokens**						
VerbToken	12.15	12.15	1	27	1.63	0.213
Group	23.97	11.98	2	27	1.61	0.219
VerbToken × Group	135.10	67.55	2	27	9.06	< 0.001***
**Motion Verb Types**						
VerbType	43.35	43.35	1	27	17.99	< 0.001***
Group	3.67	1.84	2	27	0.76	0.477
VerbType × Group	48.10	24.05	2	27	9.98	< 0.001***

‘***’ *p* < .001,

‘**’ *p* < .01,

‘*’ *p* < .05,

‘.’ *p <* .10.

**Table 6:** GLMM results for the within-language contrasts of bilinguals’ English productions compared to Turkish monolinguals and English monolinguals.

	*B*	*SE*	Wald’s *Z*	*p*
**Motion Packaging**				
Intercept	−0.17	0.34	−0.49	0.624
Group_English Monolinguals_	0.74	0.48	1.56	0.118
Group_Turkish Monolinguals_	−1.03	0.47	−2.18	0.029 *
**Motion Verb Tokens**				
Intercept	0.58	0.28	2.11	0.035 *
Group_English Monolinguals_	0.23	0.36	0.62	0.533
Group_Turkish Monolinguals_	−1.14	0.36	−3.19	0.001 **
**Motion Verb Types**				
Intercept	0.83	0.29	2.86	0.004 **
Group_English Monolinguals_	0.27	0.40	0.67	0.500
Group_Turkish Monolinguals_	−1.05	0.39	−2.73	0.006 **

* *p* < .05,

** *p* < .01,

*** *p* < .001.

**Table 7:** LMM results for the between-language contrasts of bilinguals’ Turkish productions compared to Turkish monolinguals and English monolinguals.

	SumSq	MeanSq	Num*DF*	Den*DF*	F-Value	*p*
**Motion Packaging**						
Packaging	176.82	176.82	1	54	13.33	<0.001***
Group	1.63	0.82	2	54	0.06	0.940
vPackaging × Group	229.63	114.82	2	54	8.65	<0.001***
**Motion Verb Tokens**						
VerbToken	0.15	0.15	1	27	0.02	0.896
Group	1.20	0.60	2	27	0.07	0.933
VerbToken × Group	132.10	66.05	2	27	7.71	0.002**
**Motion Verb Types**						
VerbType	20.42	20.42	1	27	9.43	0.005**
Group	1.56	0.78	2	27	0.36	0.700
VerbType × Group	48.63	24.32	2	27	11.23	<0.001***

‘***’ *p* < .001,

‘**’ *p* < .01,

‘*’ *p* < .05,

‘.’ *p <* .10.

**Table 8:** GLMM results for the within-language contrasts of bilinguals’ Turkish productions compared to Turkish monolinguals and English monolinguals.

	*B*	*SE*	Wald’s *Z*	*p*
**Motion Packaging**				
Intercept	−1.26	0.32	−3.95	<0.001***
Group_English Monolinguals_	1.83	0.45	4.02	<0.001***
Group_Turkish Monolinguals_	0.06	0.45	0.14	0.890
**Motion Verb Tokens**				
Intercept	−0.17	0.22	−0.79	0.427
Group_English Monolinguals_	0.98	0.33	2.99	0.003**
Group_Turkish Monolinguals_	−0.39	0.32	−1.22	0.222
**Motion Verb Types**				
Intercept	0.19	0.25	0.75	0.454
Group_English Monolinguals_	0.91	0.37	2.46	0.014*
Group_Turkish Monolinguals_	−0.41	0.36	−1.15	0.249

* *p* < .05,

** *p* < .01,

*** *p* < .001.

## Footnotes

1 Languages also use other means to mark manner of motion: Mandarin Chinese frequently uses a serial-verb construction in which both path and manner information are encoded in separate elements but within the bounds of a clause (e.g., Chen Guo, 2009; Ji et al., 2011), and several languages use morphemic markers (e.g., Hokan and Penutian, DeLancey, 1996; Nez Perce, Aoki, 1970) or ‘preverbs’ (Jaminjung, Schultze-Berndt, 2000) to encode manner. The marking of manner in V-languages also shows some variability, with the use of ideophones to mark manner of motion in Japanese (Hamano, 1998; Kita, 1997), Basque (Ibarretxe-Antuñano, 2004b), and Ewe (Westerman, 1930).

2 The choice to examine conflated packaging (rather than separated packaging) and manner verb (rather than path verb) bias is arbitrary, but because there are only two possible outcomes for each target motion expression (i.e., conflated/separated, manner/path) the results of one outcome level are the reciprocal of the other. By determining the likelihood of the production for a particular motion element, we can determine the relative strength of a group of speakers’ biases to prefer the expression of one motion element over another and to determine whether the strength of this bias differs between groups.

3 We tested possible order effects for packaging and lexicalization of motion using independent t-tests and found no evidence for an effect of ordering in bilinguals’ L1 Turkish or L2 English productions in either speakers’ raw or proportional production rates (all *p*’s > 0.050). We therefore collapsed the two orders in our analysis and reporting of the results.

4 Each boxplot displays the central tendency and full range of scores. Measures include the median (dark horizontal line), first through third quartiles (bottom and top of the shaded boxes, respectively), range (whiskers), and outliers (open circles).

5 We have taken a conservative approach to interpreting marginal differences between bilinguals’ Turkish (L1) productions with monolingual English speakers’ productions. As such, we suggest that these marginal differences in the planned contrasts for conflated packaging and manner verb types do *not* provide sufficient evidence that the bilinguals’ productions in L1 Turkish are comparable to the productions of English monolinguals.

## APPENDIX

See Table A and Table B.

**Table A:** Types and frequency of manner and path verbs produced in English by monolingual speakers of English and bilinguals speaking in English (L2).

Verb	English Monolinguals	Bilinguals in English
**Manner**		
catapult	1	0
chase	14	4
climb	8	6
crawl	1	0
drop	0	2
dump	1	0
escape	2	10
fly	1	0
hop	1	0
jump	2	6
kick	1	0
knock	2	0
leap	1	0
pop	11	0
pull	0	1
push	0	1
run	4	11
sneak	4	0
swarm	2	0
swim	0	1
take off	2	0
throw	5	3
tip	1	0
toss	1	0
venture	1	0
walk	6	3
wander	1	0
		
**Path**		
come	13	5
cross	1	0
fall	16	16
follow	2	1
land	0	1
leave	1	4
turn	1	1

**Table B:** Types and frequency of manner and path verbs produced in Turkish by monolinguals speakers of Turkish and bilinguals speaking in Turkish (L1).

Verb	Gloss	Turkish Monolinguals	Bilinguals in Turkish
**Manner**			
at	‘horse’	6	12
atla	‘jump’	0	1
atla-hopla	‘jump-hop’	1	0
firlat	‘throw’	0	1
gez	‘wander’	1	1
it	‘push’	2	0
kaç	‘run-away’	14	16
kaçır	‘make-run-away’	2	0
kos	‘run’	5	4
kovala	‘chase’	7	7
saldır	‘attack’	2	0
tırman	‘climb’	0	5
yarış	‘race’	1	0
yuvarlan	‘roll’	1	0
zıpla	‘jump’	1	5
			
**Path**			
ayrıl	‘leave’	2	0
cık	‘exit’	30	34
dön	‘frost’	3	4
düş	‘fall’	17	15
düşür	‘drop’	4	2
geç	‘pass’	1	0
gel	‘come’	8	3
gir	‘enter’	2	3
ilerle	‘advance’	0	1
in	‘descend’	1	0
takipet	‘follow’	1	0
terket	‘leave’	1	0
uzaklaş	‘move-away’	2	0
yönel	‘head-towards’	1	0
